# Coracoid bending for refractory anterior upper arm pain after reverse total shoulder arthroplasty

**DOI:** 10.1016/j.xrrt.2026.100750

**Published:** 2026-04-09

**Authors:** Naoko Mizuno, Yuki Kotani, Shigeto Nakagawa, Kenji Hayashida

**Affiliations:** a Department of Orthopaedic Surgery, Toyonaka Municipal Hospital, Osaka, Japan; b Department of Sports Orthopaedic Surgery, Yukioka Hospital, Osaka, Japan; c Department of Orthopaedic Surgery, Osaka Central Hospital, Osaka, Japan

**Keywords:** Reverse total shoulder arthroplasty, Coracoid bending, Anterior upper arm pain, Coracoid process, Conjoint tendon, Humeral distalization

## Introduction

Reverse total shoulder arthroplasty (rTSA) reliably provides favorable clinical outcomes for patients with severe rotator cuff deficiency, resulting in substantial pain relief and improved shoulder function. However, medialization and distalization of the glenohumeral center of rotation increase deltoid tension and impose continuous elongation forces on the conjoint tendon, which is composed of the short head of the biceps and the coracobrachialis and originates from the coracoid process. Excessive tension on this tendon has been suggested as a potential cause of localized coracoid tenderness and deep anterior upper arm pain. Accordingly, persistent anterior shoulder or upper arm pain may occur even in the absence of infection, implant loosening, instability, or fracture.^1^^,^^2^^,^^4^^,^^5^^,^^8^, ^9^, ^10^

We present a case in which we considered excessive conjoint tendon tension due to humeral distalization to be the primary source of pain. We performed a novel technique involving coracoid bending, glenosphere modification, polyethylene exchange, and pectoralis minor release to reduce this tension while preserving native anatomy.

### Case presentation

A 71-year-old woman (height: 143 cm) with a history of rheumatoid arthritis, which was diagnosed in her 40s, presented with persistent pain in her right upper arm and limited function. Eight years earlier, she underwent primary rTSA for a massive rotator cuff tear. A Grammont-type prosthesis (Aequalis Reversed II, Tornier, France) was implanted; however, due to the small size of the proximal humerus, adequate metaphyseal bony containment could not be achieved. She continued to experience persistent anterior upper arm pain post-operatively, and active shoulder elevation was impossible due to the pain.

Two years after the initial surgery, she developed posterior shoulder pain, which was attributed to irritation of the suprascapular nerve by the superior and posterior glenoid screws. Although removal of these screws resolved the posterior pain, anterior shoulder pain localized to the coracoid region persisted, and active elevation remained severely restricted.

**Figure 1:**
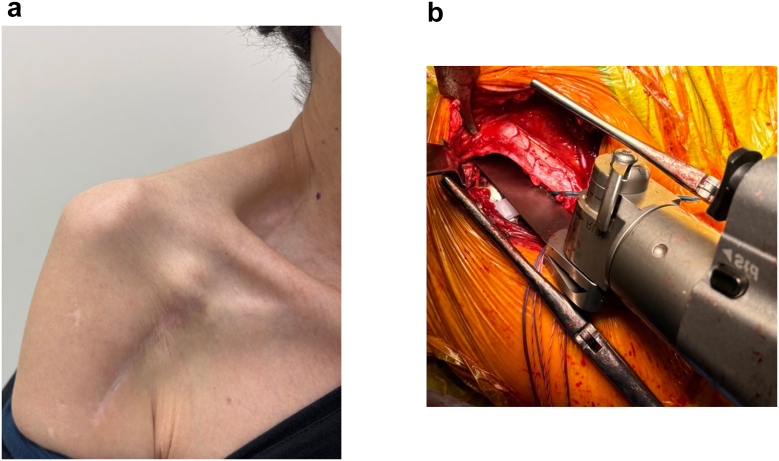
Pre-operative findings and coracoid preparation. (**a**) Pre-operative clinical photograph showing marked deltoid atrophy, prominence of the coracoid process, and increased tension of the pectoralis minor. (**b**) Intraoperative photograph showing the creation of a bone trough at the "elbow" of the coracoid by selectively removing the inferior cortical surface with a bone saw.

Physical examination revealed marked deltoid atrophy due to prolonged disuse (Fig. 1*a*). No motor weakness or sensory disturbance was observed. Although anterior deltoid atrophy raised suspicion of axillary nerve palsy, needle electromyography revealed no evidence of axillary nerve or brachial plexus dysfunction. Furthermore, no clinical findings were suggestive of quadrilateral space syndrome. There was no evidence of thoracic kyphosis, and the scapulae were positioned symmetrically on both sides. There was no clinically relevant scapular malposition, protraction, internal rotation, or scapulothoracic dysfunction. She reported pain localized to the coracoid process that extended distally along the conjoint tendon. Tenderness was localized to the coracoid, conjoint tendon, and pectoralis minor, with radiating pain along the course of the conjoint tendon. Active forward elevation was limited to 10°, whereas passive elevation reached 140°. External rotation was 0° at the side, while internal rotation reached the ipsilateral thigh. The pre-operative Constant score was 10 points. A diagnostic local anesthetic block of the proximal conjoint tendon resulted in significant pain relief. After the injection, she could actively elevate her arm, which had been impossible before due to pain. Due to the limited volume and intratendinous location, it was considered unlikely that the anesthetic effect would extend to the brachial plexus, and no transient motor or sensory changes were observed. In the absence of infection or instability, excessive humeral distalization with increased conjoint tendon tension was considered the primary pathology. Therefore, coracoid bending with glenosphere modification, polyethylene exchange, and pectoralis minor release was planned.

### Surgical technique

The procedure was performed with the patient in the beach-chair position and using the deltopectoral approach previously employed. After exposing the coracoid process, we found that the conjoint tendon was adherent to the surrounding tissues and appeared markedly tense.

**Figure 2:**
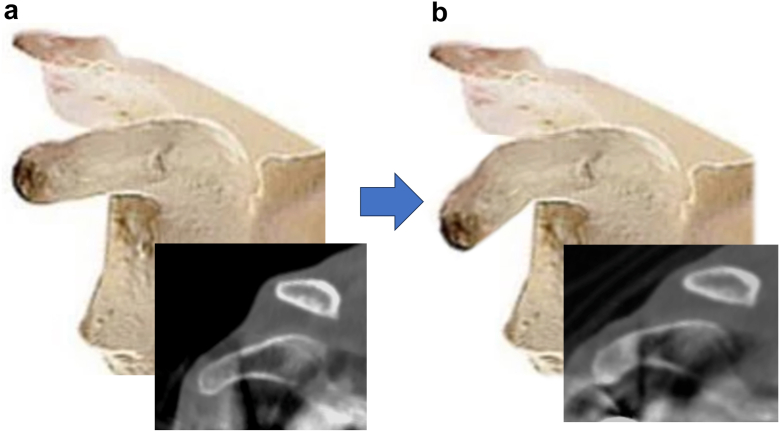
Coracoid bending procedure. Schematic illustrations and computed tomography images obtained before and after the coracoid bending procedure. (**a**) Pre-operative appearance. (**b**) Post-operative appearance which demonstrates controlled caudal bending of the coracoid process while preserving the continuity of the superior cortical bone.

A bone trough was created at the "elbow" of the coracoid by selectively removing the inferior cortical surface with a bone saw while preserving the superior cortex as a hinge (Fig. 1*b*). After creating the trough, the coracoid was gently bent caudally with slow, controlled manual pressure applied by the surgeon's finger (Fig. 2). This maneuver resulted in an immediate and visible reduction in tension of the conjoint tendon. Excessive force must be avoided during coracoid bending to preserve the superior cortical hinge; loss of this hinge may increase the risk of coracoid fracture.

**Figure 3:**
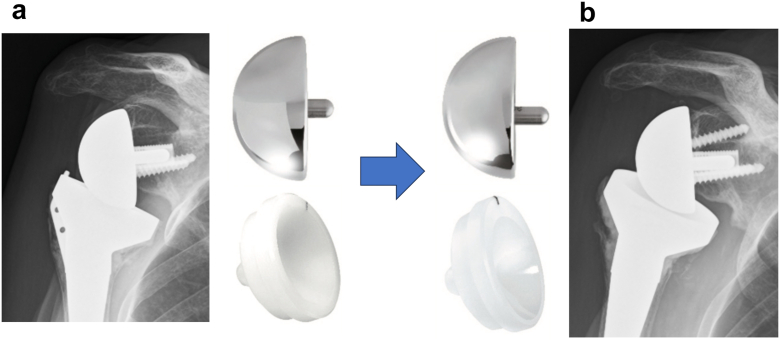
Implant modification to reduce humeral distalization. Pre-operative and post-operative plain radiographs demonstrating changes in the glenosphere position and humeral insert configuration. (**a**) Pre-operative radiograph showing a standard implant configuration. (**b**) Post-operative radiograph showing implantation of an eccentric glenosphere in a superior offset position and exchange to a lateralized humeral insert.

Combined procedures were performed. The pectoralis minor tendon, which was noted to be tight, was released to reduce anterior soft-tissue tension further. Then, the previously removed superior and posterior glenoid screws were reinserted. To address excessive humeral distalization, an eccentric glenosphere with superior offset was implanted, and the humeral insert was exchanged for a lateralized component (Fig. 3).

### Imaging findings

Pre-operative radiographs showed that the metaphyseal portion of the humeral stem was positioned proximal to the humeral osteotomy line. Humeral distalization measured 26 mm compared to the pre-rTSA state. There were no signs of implant loosening. The superior edge of the glenosphere was located 1.7 mm distal to the superior glenoid rim (Fig. 3*A*). Post-operative radiographs revealed a 3.3 mm proximal shift of the superior edge of the glenosphere, now positioned 1.6 mm proximal to the superior glenoid rim (Fig. 3*b*).

### Post-operative course

**Figure 4:**
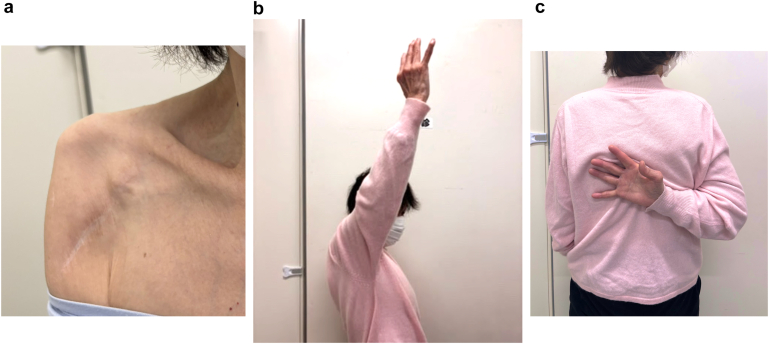
Post-operative clinical outcome. (**a**) Post-operative clinical photograph showing resolution of coracoid prominence and reduction of pectoralis minor tension. (**b** and **c)** There are marked improvement in shoulder function, with active forward elevation reaching 150° and internal rotation reaching the T8 vertebral level.

The arm was immobilized in a sling for two weeks. Range-of-motion exercises were started one week after surgery. Pain improved rapidly. Three months post-operatively, the visual analog scale score decreased from 8 to 1, and active forward elevation improved to 130°. At the final follow-up, 25 months after surgery, active forward elevation improved further to 150°. External rotation at the side reached 30°, and internal rotation improved to the T8 vertebral level. The Constant score increased to 81 points (Fig. 4).

## Discussion

In rTSA, medialization and distalization of the center of rotation enhance deltoid efficiency but simultaneously impose continuous elongation forces on the conjoint tendon that arises from the coracoid process. This altered soft-tissue tension can lead to localized coracoid tenderness and deep anterior upper arm pain.^1^^,^^2^^,^^4^^,^^5^^,^^8^, ^9^, ^10^ Ekelund et al^2^ emphasized that persistent pain after rTSA should prompt consideration of soft tissue–based pathologies, including altered muscle tension, adhesions, and subcoracoid impingement, as well as implant-related causes.

Hao et al^5^ reported that humeral distalization exceeding 25 mm is associated with an increased risk of pain and neurological symptoms. In the present case, humeral distalization measured 26 mm. Tenderness was localized to the coracoid, conjoint tendon, and pectoralis minor. Radiating pain was present along the conjoint tendon. These findings support the idea that excessive soft-tissue tension is the primary source of pain.

The literature has reported nerve injury after rTSA, with the axillary nerve being the most frequently involved.^7^ One possible contributing mechanism is excessive distalization. Rapid pain relief following local anesthetic injection has also been observed in compressive neuropathic conditions. In this case, however, no clinical or electrophysiological evidence of axillary nerve or brachial plexus dysfunction was identified. Therefore, the immediate response was considered to be more consistent with the relief of anterior soft-tissue tension. However, a neuropathic component cannot be entirely excluded.

Recent evidence has demonstrated that scapulothoracic orientation significantly influences clinical outcomes after rTSA, particularly with respect to post-operative range of motion and functional recovery.^6^ In this case, no thoracic kyphosis was observed, and the scapulae were positioned symmetrically on both sides. No clinically relevant scapular malposition, protraction, internal rotation, or scapulothoracic dysfunction was identified. Accordingly, scapulothoracic malalignment was considered unlikely to be a primary contributor to the patient's symptoms.

Several surgical treatments for anterior shoulder and upper arm pain after rTSA have been described. These treatments primarily target excessive tension or impingement of the conjoint tendon and surrounding soft tissues. Friedman et al^3^ validated the “human disharmony loop” concept by demonstrating that pectoralis minor tenotomy reduces pain and improves function in certain patients. Although our case does not strictly meet their criteria, it reinforces the potential role of anterior soft-tissue tension in persistent shoulder pain. Tashjian et al^10^ reported that releasing the conjoint tendon alone provided meaningful pain relief in patients with persistent anterior shoulder pain following rTSA without major complications, supporting the concept that excessive tension in the conjoint tendon may function as a primary pain generator in certain cases. Similarly, Gómez et al described Z-lengthening of the conjoint tendon as an effective method for relieving symptoms in a series of treated patients; however, this technique depends on biological healing and alters tendon continuity.^4^ From a biomechanical perspective, Qawasmi et al^8^ demonstrated in a cadaveric study that conjoint tendon lengthening improves internal rotation after rTSA, further supporting the idea that adjusting anterior soft-tissue tension can positively affect post-operative shoulder function.

Other authors have focused on subcoracoid pathology and local impingement as potential sources of pain. Ardebol et al^1^ reported favorable outcomes following arthroscopic management for anterior shoulder pain and stiffness after rTSA, including débridement and procedures that address subcoracoid impingement, such as limited coracoid bone resection or partial coracoidplasty. Satalich et al^9^ described arthroscopic coracoid excision combined with conjoint tendon and pectoralis minor release as a treatment option for refractory anterior shoulder pain after rTSA. Although these procedures can be effective, tendon release can compromise elbow flexion strength. Tendon lengthening depends on biological healing, and coracoid resection alters native anatomy. In contrast, coracoid bending is designed to reduce excessive tension in the conjoint tendon without sacrificing the tendon or removing substantial bone. A partial osteotomy of the inferior cortex combined with controlled distal bending of the coracoid while preserving the superior cortical hinge reduces tension while maintaining anatomical integrity and muscle–tendon continuity.

In this case, the intraoperative findings confirmed an immediate reduction in conjoint tendon tension after the coracoid bending. Post-operative pain relief and functional improvement occurred rapidly. However, multiple surgical adjustments were performed, including glenosphere modification, polyethylene exchange, and pectoralis minor release. There is literature supporting the potential contribution of each of these interventions to symptom resolution. Therefore, the observed improvement was multifactorial and cannot be primarily attributed to the coracoid bending procedure. Modification of coracoid morphology remains a hypothesis-generating concept. The present case represents a mechanism-based clinical observation rather than definitive evidence supporting the routine use of this procedure. Larger, controlled studies are needed to clarify its independent contribution. In addition to its use in the post-operative setting, this technique may also be applicable intraoperatively when excessive tension of the conjoint tendon is encountered after reduction and implant positioning during rTSA. In such situations, coracoid bending could serve as an adjunctive option to immediately adjust soft-tissue tension and optimize balance during surgery before closing the wound. Although the present procedure was performed through an open deltopectoral approach, the technique may be adaptable to a fully arthroscopic approach. With appropriate visualization and specialized instrumentation, selective inferior cortical osteotomy of the coracoid followed by controlled bending could potentially be achieved, offering a minimally invasive option for managing refractory anterior upper arm pain following rTSA.

## Conclusion

Coracoid bending may be a useful surgical option for patients with excessive conjoint tendon tension due to humeral distalization as the primary source of pain. This technique, which preserves anatomy, allows adjustment of anterior soft-tissue tension. It may be used as a post-operative salvage procedure for refractory anterior upper arm pain after rTSA or as an intraoperative adjunctive option when excessive anterior soft-tissue tension is encountered.

In this case, however, the clinical improvement resulted from multiple combined surgical interventions, so the independent contribution of coracoid bending cannot be definitively determined. Further investigation is necessary to clarify its effect when used alone.

## Declaration of generative AI and AI-assisted technologies in writing process

During the preparation of this manuscript, the authors used ChatGPT (OpenAI) for minor language editing. The scientific content, data interpretation, and conclusions were developed solely by the authors. The authors reviewed and approved the final version and take full responsibility for the manuscript.

## Disclaimers:

Funding: No funding was disclosed by the authors.

Conflicts of interest: The authors, their immediate families, and any research foundations with which they are affiliated have not received any financial payments or other benefits from any commercial entity related to the subject of this article.

Patient consent:Informed consent for publication was obtained from the patient.
